# Measurements of Energetic States Resulting from Ion Exchanges in the Isomorphic Crystals of Apatites and Bioapatites

**DOI:** 10.3390/molecules27248913

**Published:** 2022-12-15

**Authors:** Andrzej Kuczumow, Tomasz Blicharski, Mieczysław Gorzelak, Jakub Kosiński, Agnieszka Lasota, Jacek Gągała, Jakub Nowak, Maciej Jarzębski, Mirosław Jabłoński

**Affiliations:** 1 Lab 196, Radawiec Duży 196, 21-030 Motycz, Poland; 2 Department of Orthopaedics and Rehabilitation, Medical University of Lublin, K. Jaczewskiego 8, 20-090 Lublin, Poland; 3 Chair and Department of Jaw Orthopedics, Medical University of Lublin, Chodźki 6, 20-093 Lublin, Poland; 4 Department of Orthopaedics and Traumatology, Medical University of Lublin, K. Jaczewskiego 8, 20-090 Lublin, Poland; 5 Department of Physics and Biophysics, Poznan University of Life Sciences, Wojska Polskiego 38/42, 60-637 Poznan, Poland

**Keywords:** apatites, bioapatite, X-ray diffraction, energy changes, ion exchanges

## Abstract

Developments in the field of nanostructures open new ways for designing and manufacturing innovative materials. Here, we focused on new original ways of calculating energy changes during the substitution of foreign ions into the structure of apatites and bioapatites. Using these tools, the energetic costs of ion exchanges were calculated for the exemplary cases known from the literature. It was established that the most costly were ion exchanges of some cations inside apatites and of anions, and the least costly exchanges in tetrad channel positions. Real energy expenses for bioapatites are much smaller in comparison to mineral apatites due to the limited involvement of magnesium and carbonates in the structure of hard tissues. They are of the order of several electron volts per ion. The rigorous dependences of the energy changes and crystallographic cell volumes on the ionic radii of introduced cations were proved. The differentiation of the positioning of foreign ions in locations of Ca(I) and Ca(II) could be calculated in the case of a Ca-Pb reaction in hydroxyapatite. The energetic effects of tooth aging were indicated. The ability of energy change calculation during the ion exchange for isomorphic substances widens the advantages resulting from X-ray diffraction measurements.

## 1. Introduction

Substances called bioapatites are specific materials that are formed in living organisms as a basic part of hard tissues. The word apatite arrives in their names to emphasize that they are strictly related to mineral hydroxyapatite and other similar apatites but by no means identical ones [1,2,3,4]. From a chemical point of view they differ by the presence of such ions as Mg^2+^ and Na^+^ as cationic entities, CO_3_^2−^ and HPO_4_^2−^ as anions, and, to a lesser degree, K^+^ and Sr^2−^. Amounts of the hydroxyl ion OH^−^ are smaller than in the original hydroxyapatite. The degree of crystallinity is lower than in hydroxyapatite. Since the arrival of vertebrates, nature has adapted apatite as a base for the construction of their hard tissues. Thus, detailed knowledge of bioapatites and apatites is very important. To be right, we must add that geological apatites have ionic admixtures as well, and we try to compare bioapatites rather than an idealized pure substance with the rigorous formula: Ca_10_(PO_4_)_6_(OH)_2_. The apatites, both in biological and mineralogical versions, have the rather unusual feature of being susceptible to intensive ion exchanges [5,6] without an essential change in structure. With the change in the structure, one should consider the conservation of the crystallographic system, but not the dimensions, as is characteristic for isomorphic substances. Different degrees of ion exchanges are possible, from rather limited (Mg^2+^, CO_3_^2−^) to nearly total (Sr^2+^, Ba^2+^, Pb^2+^, Cd^2+^, F^−^, OH^−^). According to many announcements, the liquid surface layer is responsible for the extensive ion exchanges [7]. Due to the different sizes of exchanged ions, possible defects, and vacancies, the dimensions of crystallographic cells of substances differ in some values. If precisely measured in diffraction experiments, they can be an invaluable source of information about the structure of cells.

Similarly, foreign ions enter the apatite structure not only as relatively small admixtures, but they can also simply be the main components of apatites, e.g., when somebody replaces Ca with Sr or Ba, phosphates with vanadates or arseniates, or OH^−^ with Cl or F [8]. Such new systems we observe in geological samples, sometimes as solid solutions series [9]. Finally, material scientists try to prepare artificial apatites [10] with differently tailored admixtures as synthetic biomaterials for stomatological or orthopaedical aims. Sometimes, the syntheses have a biomimetic character [11].

Scientists study the bioapatite and apatites with chemical analysis (mainly with Electron Probe Microanalysis EPMA [12,13], Fourier-transform infrared (FTIR) spectroscopy and microscopy [14,15,16,17], and/or Raman spectroscopy and microscope techniques [18,19]), structural analysis (X-ray diffraction (XRD) [20,21] and/or neutron diffraction [22]), or morphological points of view (scanning electron microscopy (SEM), transmission electron microscopy (TEM) [23], or atomic force microscopy (AFM) [24]). In addition, some energetic or thermodynamic data are collected, based mainly on thermogravimetric measurements [25,26]. The studies on energetic conditions for ion exchange are relatively scarce. The ignition of apatite biomaterials or the synthetic components leading to the formation of imitations of bio/apatites is another subject of energetic character that has been studied [27]. Finally, the high-temperature phase diagrams for the system CaO-P_2_O_5_ were presented [28]. The authors of recent contributions have become convinced that by using the diffraction methods we can obtain valuable information about the energy of crystallographic transformations at the crystallographic cell level.

The main aims of the recent contribution are to derive the relevant original formulae concerning the energy changes during the crystal transformation at the cellular level. Furthermore, our study focused on comparing the energy changes occurring during stepwise ionic exchanges in apatites. Finally, the study estimates the energy variability during transformations of biological apatites connected with the ion exchanges.

## 2. Results

We used the idealized formula for hydroxyapatite Ca_10_(PO_4_)_6_(OH)_2_ as a reference point for illustrating the energetic changes during the conversions of hydroxyapatite in substituted forms. The variability of cations (shown in black in the figures), anions (red), and ions existing in the tetrad channel (blue) will be considered. The results will be shown as a series of particular cases.

### 2.1. Theory

We consider here the hexagonal structure as the one relevant for the apatites. One can calculate the energy difference at the particle level for the molecular dimension d. This dimension is good for this aim, since it involves contributions from “a” and ”c” dimensions. We use a specific form of Braggs law, where the wavelength is substituted by energy with the proper coefficient:

n ∗ 12.4/E = 2d ∗ sinΘ(1)

with universal dimension d. Using Miller indices (hkl) for hexagonal system:

1/d^2^ = 4/3(h^2^ + hk + k^2^)/a^2^ + l^2^/c^2^ (2)

we can split the value of “d” on components “a” and “c”. Introducing n = 1 we can calculate the energy for some lines:

6.2/d_1_sinΘ_1_ = E_1_ (3)

For the same line in another sample, which is slightly shifted, we can write:

6.2/d_2_sinΘ_2_ = E_1_ (4)

Although the energy of exciting radiation is constant, we can still ask what happens when we put the value of d_1_ into Equation (4). In fact, one seeks the energy shift ΔE in exciting radiation, which will allow the fulfilment of Braggs law.

ΔE = (6.2/d_1_)(1/sinΘ_1_ − 1/sinΘ_2_) (5)

One can make inverse reasoning, namely what it means when we introduce a constant angle function (sinΘ) to Equation (3). It is equivalent to a recognition that the energy difference results from the variability in dimension “d”. The inverted order in coefficients and the following:

ΔE = (6.2/sinΘ_1_)(1/d_2_ − 1/d_1_) (6)

result from the necessity of keeping the same sign of the expression.

The problem of energy shift can be solved in another way. Equation (3) can be differentiated (setting sinΘ as a constant) and passing from differentials to differences:

ΔE = −6.2 Δd /(d^2^ sinΘ) (7)

or, recognizing “d” as a constant:

ΔE = −6.2 Δ(sinΘ)/(d sin^2^Θ) (8)

How can it be proven? Equation (5) can be approximated to Equation (8):

ΔE = (6.2/d_1_)(1/sinΘ_1_ − 1/sinΘ_2_) = (6.2/d_1_)(1/sinΘ_1_ − 1/(sinΘ_1_ + Δ(sinΘ )) ≈ −6.2 Δ(sinΘ)/(d_1_ sin^2^Θ_1_) (9)

Similarly, one can prove in the same trivial way that Equation (6) is approximately equivalent to Equation (7).

Finally, by a somewhat different differentiation of Equation (3) as a function of E and d, with sinΘ as a constant, one can obtain:

ΔE = −(1/6.2) ∗ Δd E^2^ sinΘ (10)

Equations (8) and (10) are joined as the reciprocals. If one compares the right sides of both equations, the resulting equation can be reduced to Equation (1).

If somebody is interested in the calculation of energies of transformations along particular axes “a” and “c”, then one must invoke Equation (2). In further calculations in this text, we use hkl indices set as (1,1,1). It takes into account the contributions from changes along dimensions “a” and “c” in possibly the simplest way.

A simple check was done on the results by Dorozhkin [29] for his data concerning the passing from dentin to enamel. Using Equations (5)–(10) we had, respectively: −8.52 eV; −8.75 eV; −8.52 eV, −8.51 eV, and −8.52 eV as the energy changes. The calculations show a very uniform set of results and full convertibility of the equations. One can observe as an interesting fact that if we compare the right sides of Equations (7) and (10), than we derive Equation (1).

It is obvious that Equations (5)–(10) can be applied exclusively for the same types of chemical compounds and the same type of crystallographic system. The interpretation of the data is that the energy differences cover changes in composition, the occurrence of vacancies, the structural deviations from the ideal positions, the change of orientation leading to smearing of reflexes, and the increased frequencies of oscillations around ideal positions. The studies on real tissues (e.g., enamel, bone) allow for an inclusion of the influence of the texture as well.

### 2.2. Transformations of Mineral Apatites

**Figure 1:**
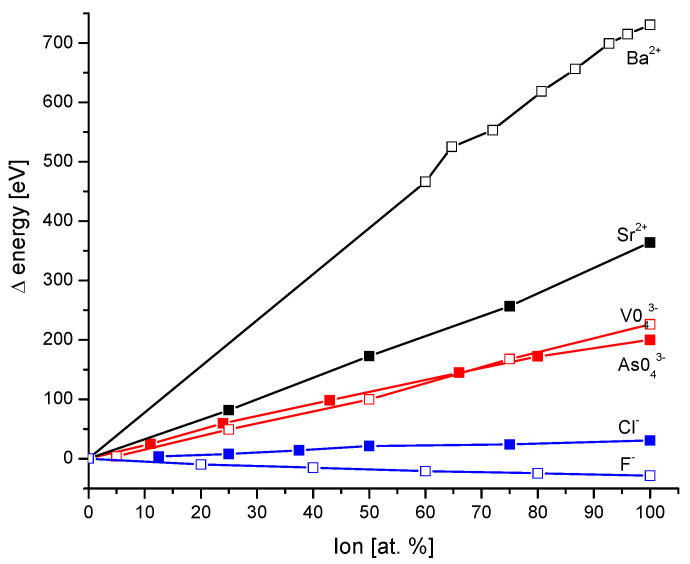
Energy changes during cationic substitutions (black), anionic substitutions (red), and substitutions in channel positions (blue) of apatites. Figure based on data presented in: for F^-^ from [35], for Cl^−^ from [34], for AsO_4_^3−^ from [30], for VO^3−^ from [31], for Sr^2+^ from [32], and for Ba^2+^ from [33] positions.

The transformation of hydroxyapatite into 100% arsenate apatite (johnbaumite) [30] demands a huge energy of 200.2 eV; similarly, the transformation of hydroxyapatite in vanadate apatite [31] is another case of the ion exchange in anion group. The transformations of hydroxyapatite in Sr-apatite [32] or Ba-apatite [33] are cases when the cation is exchanged and they are associated with even greater energies. The involvement of Cl [34] and F [35] occurs in the tetrad channel with relatively moderate energy changes (Figure 1). The curves for particular ion exchanges are more or less straight lines. The positive values of ΔE mean that the derivatives of hydroxyapatite involving foreign ions have smaller energies than the parent substance and the energy is liberated. The situation is opposite for fluorapatite.

### 2.3. Transformations in Bioapatites

**Figure 2:**
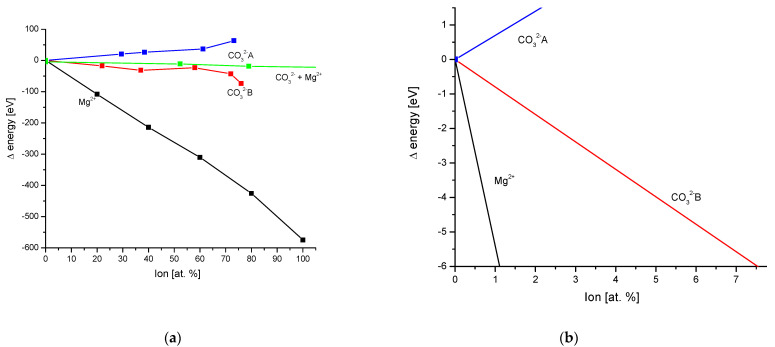
(**a**) Substitutions in bioapatites; green line presents mixed substitution of Mg and carbonates; (**b**) real biological substitutions, limited by the true ionic concentrations of Mg and carbonates. Origin of basic data: Mg^2+^ from [36], Mg^2+^ + CO_3_^2−^ from [40], CO_3_^2−^, substitution A from [39], CO_3_^2−^, substitution B from [38].

Figure 2a shows the energy added or released in bioapatites. The processes involve cationic Mg addition [36] (although, Terpstra and Driessens [37] had somewhat of another opinion about the possibilities of exchange in a whole range of concentrations) and variability of carbonate contents as anion exchange (substitution B) [38] or changes in the tetrad channel (substitution A) [39]. A separate line shows the situation when Mg and carbonates are substituted in parallel, according to Sader et al. [40]. One can see from the latter paper that the B-type substitution of CO_3_^2−^ exerts the dominating influence, as was suggested by Elliott [41]. Mg seems to not seriously influence the energy loss curves when it is associated with carbonates. Since the substitutions of the above ions are limited in real hard tissues, we estimate in Figure 2b the real level of ion exchanges and the real small range of energy variability (+1 eV down to −6 eV), with both the release (CO_3_^2−^A) and consumption of energy. The energy exchanges reach 500 kJ/mol in these cases. If one would like to use light to deliver energy to biomimetic reactions of ion exchange of Mg and CO_3_^2−^B, wavelengths up to 250 nm would be applicable.

### 2.4. Other Interesting Ion Exchanges

**Figure 3:**
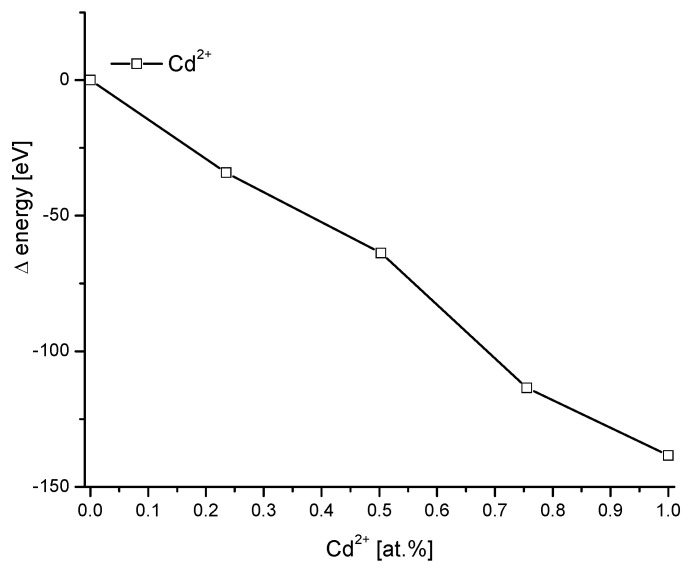
Substitution of Cd into hydroxyapatite, data from [42].

Cd is another element which can be substituted in hydroxyapatite in a wide range. We used the data from Bigi et al. [42] The results are presented in Figure 3. They are similar to those from Figure 2 for Mg; however, the value of energy changes is more moderate. The energy change is in between Mg from one side and Sr and Ba from another side. The energy must be delivered to the system in this case.

**Figure 4:**
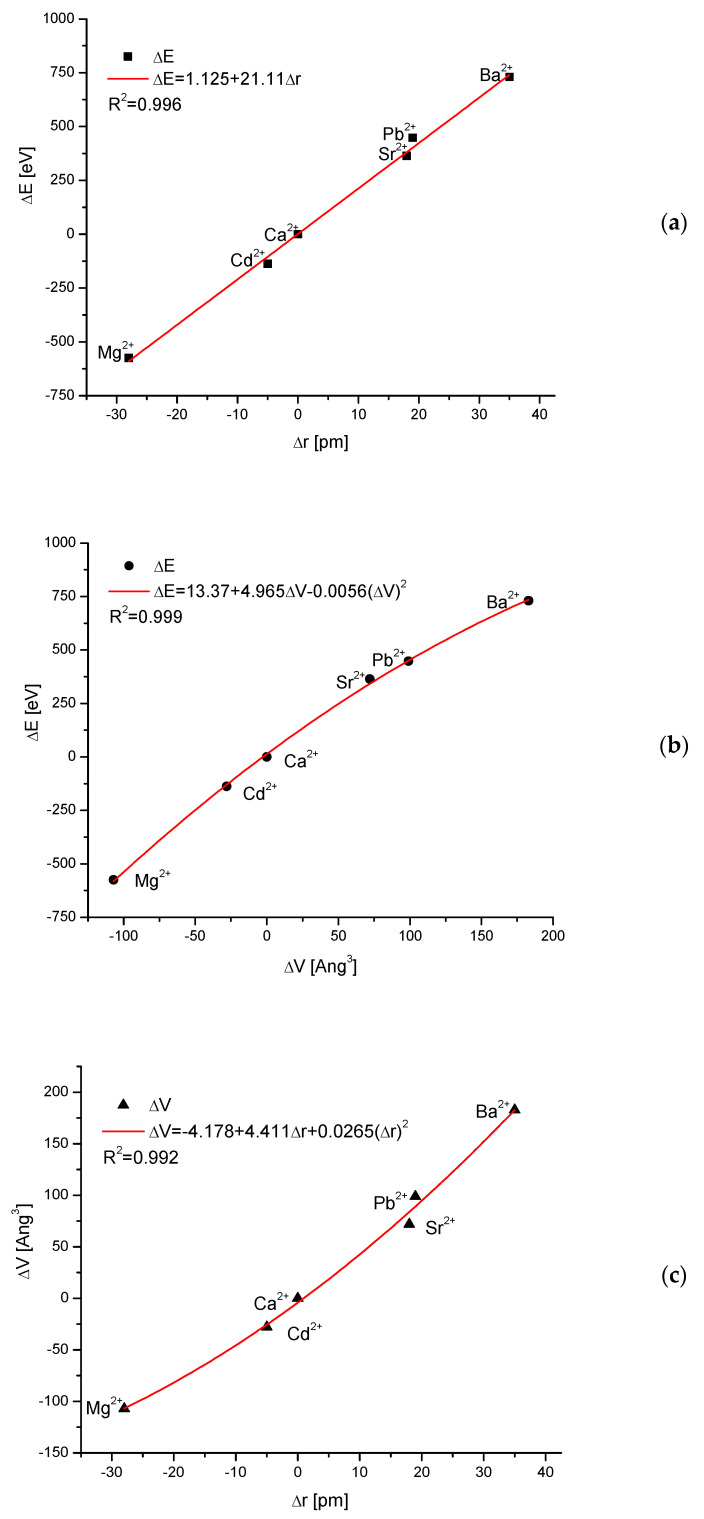
Relationship between the energy changes and the kind of introduced ions as a result of cationic substitutions in apatites; (**a**) the dependence on the difference in ionic radius of introduced ions; (**b**) on the difference in crystallographic cell volume. (**c**) The proportionality between growth of ionic radius of introduced ion and growth of crystallographic cell volume.

One can collect the maximum energies connected with the total exchange of hydroxyapatite into an apatite with new main cation (total cationic ion exchange). It can be compared with the differences between the ionic radius of the new cation and the ionic radius of Ca^2+^. It is shown in Figure 4. The result is to some degree intuitively expected, but the perfectness of the relationship is greatly surprising. Simply put, the change of energy involved in the ion exchange is directly proportional to the difference in ionic radii:

ΔE = 1.125 + 21.11Δr(11)

where r is ionic radius [43]. The coupling is linear and not more complicated. One should rather intuitively expect that this dependence would be quadratic or tertiary even due to the steric reasons. The result can be compared with Equations (5)–(10). Somewhat different is the relationship between the difference in energy and the change in the volume of the crystallographic cell–it is the second order polynomial dependence, with very small correction of second order.

ΔE = 13.37 + 4.965ΔV − 0.0056(ΔV)^2^ (12)

Finally, we proved that it is a proportional growth of crystallographic cell volume together with the growth of ionic radius of introduced cation. It can be expressed by the second order polynomial, but with very small second order correction.

ΔV = −4.178 + 4.411Δr + 0.0265(Δr)^2^ (13)

We should observe that the results shown in Figure 4 extend outside the values indicated by Goldschmidt rule. Probably this result can be extended to all cases of ion exchanges of cations in isomorphic crystallographic systems.

### 2.5. Transformations Due to Aging of Teeth

**Figure 5:**
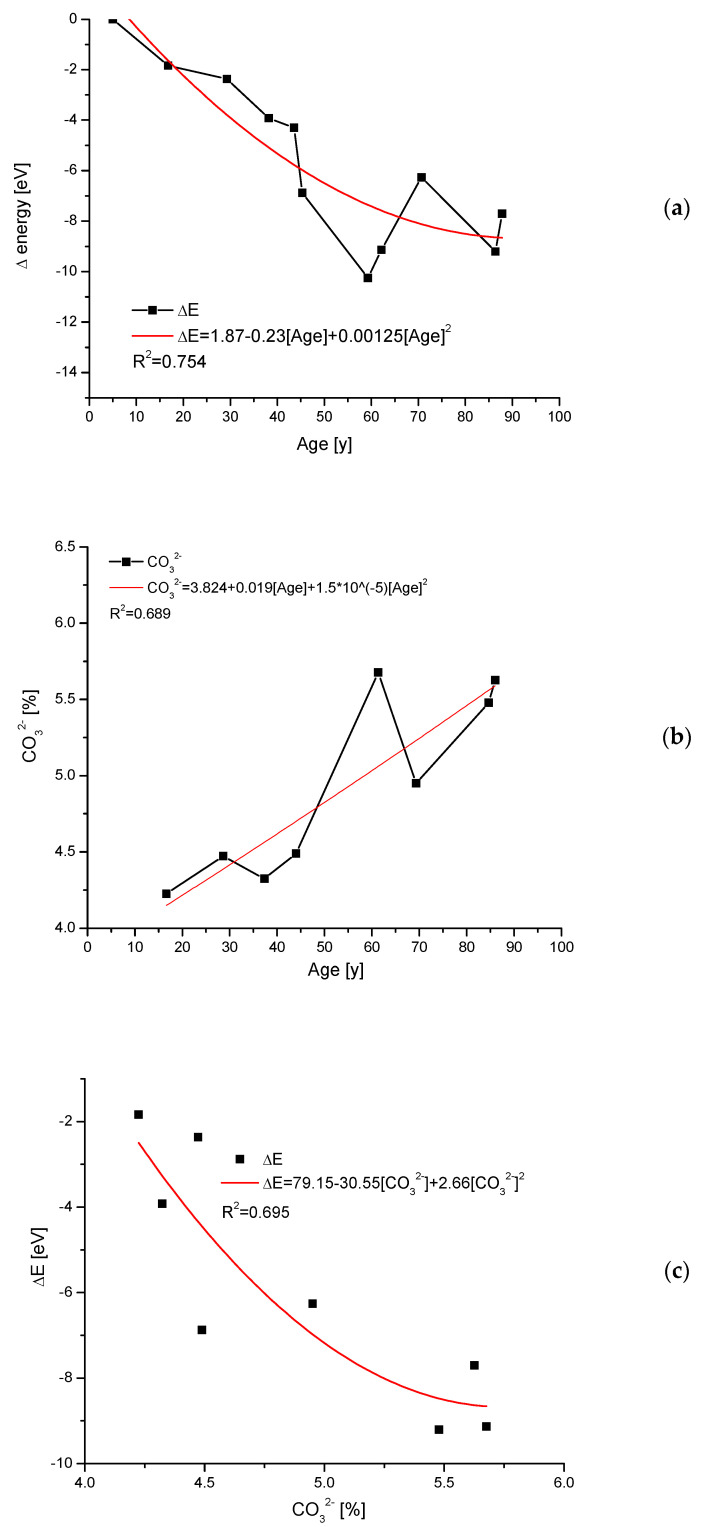
(**a**) The drop of energy change during the aging of teeth; (**b**) variability of carbonate amounts coupled with the aging; (**c**) intercoupling of energy changes with contents of carbonates. Basic data from position [45].

This variability of crystallographic dimensions (coupled in our approach with energy spending) is described, e.g., in papers by Handschin et al. [44], and, in this case, it concerns the influence of age on the status of bones. In Figure 5, the influence of aging on the tooth apatite material is shown, based on data by Leventouri et al. [45]. One can observe the increase in the energetic level of older teeth (Figure 5a) in comparison with younger ones, and it is equivalent to the increasing instability of material. At the same time, a clear increase in carbonate contents occurs (Figure 5b). If we compare this with Figure 2a, the observed changes can be associated with substitution B of carbonates. There is a spread in the results from Figure 5a,b, which translates into the value of the correlation coefficient for the relationship ΔE–age. Indeed, only moderate polynomial accordance can be observed (Figure 5c). Still, it is a good result for biological samples.

### 2.6. Energy Change during Substitutions in Ca(II) or Ca(I) Positions

**Figure 6:**
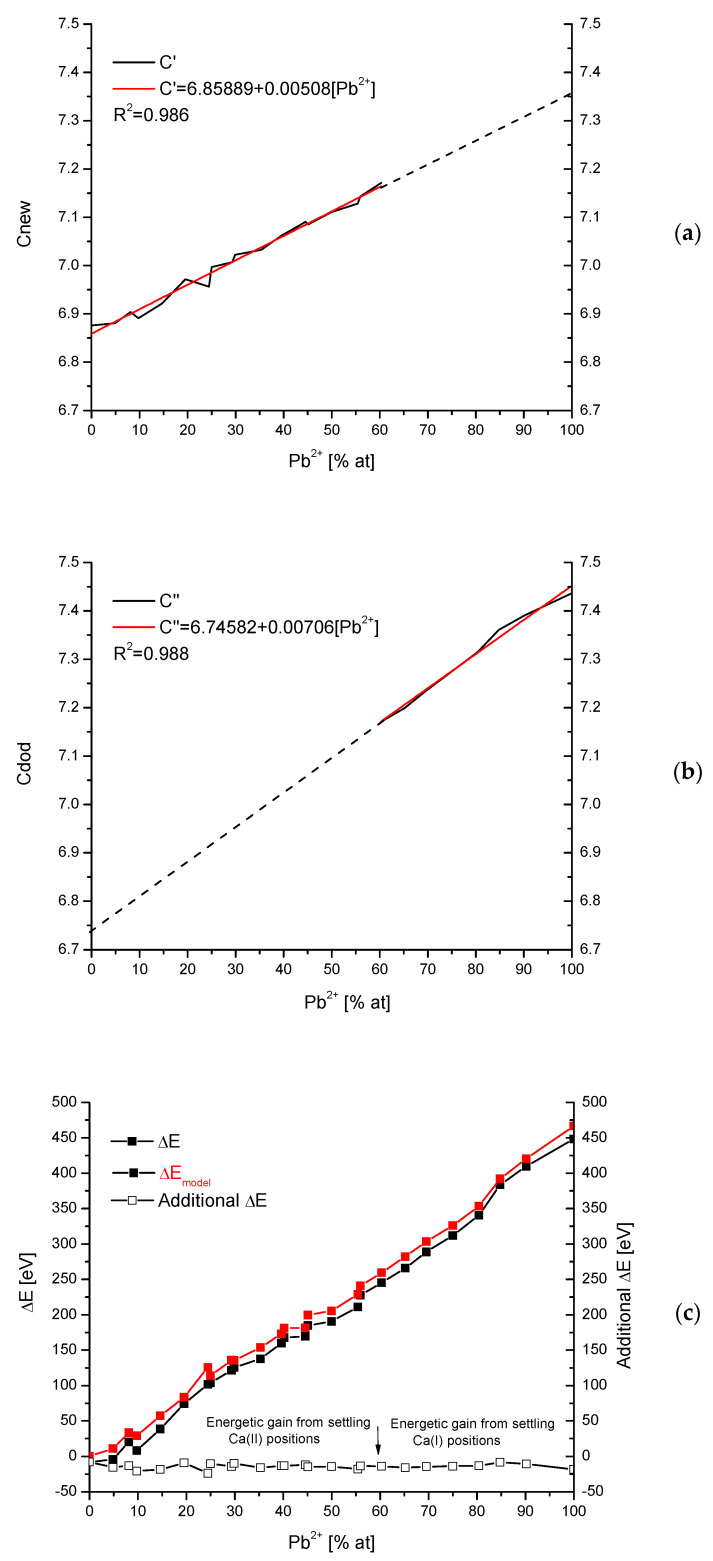
(**a**) Approximation and hypothetical prolongation of the first increment of “c” values for Ca-Pb hydroxyapatite system; (**b**) the same for second increment; (**c**) energy loss spectrum for Ca-Pb substitutions in hydroxyapatite, real (black) and hypothetic (red) one, and the spectrum of energy differences (open squares) resulting from the energetic nonequilibrium of positions Ca(I) and Ca(II). Basic data from positions [46,47].

In some cases, relatively close to solid solutions, we have single discontinuities. One can observe this, e.g., in the hydroxyapatite substituted with Pb [46,47]. One can assume that the first line approximating the “c” parameter in the zone of lower concentrations of Pb can be prolonged up to transection, with the ordinate for the concentration of 100 at%. The prolongation shows what would happen if it manages to continue the substitution in positions of ions Ca(II) (Figure 6a). Figure 6b shows a similar situation with the approximation of parameter “c” for higher values of Pb concentration. This time the prolongation of the curve up to the lowest values shows the hypothetical value of “c” for the situation as if the substitution of Ca(I) positions would be preferential in this range. We can extract the postulated values of the “c” parameter in the whole range, add the real values of “a” (it does not reveal the discontinuity), and calculate the hypothetical loss of energy spectrum for the system of Ca-Pb hydroxyapatites. Of course, for real values of “a” and “c”, we have another real spectrum of energy losses. If we subtract one spectrum from the other, we obtain the spectrum of additive energy that would be necessary to perform the synthesis in such a way that Ca(I) ions are substituted first. It is worth noticing that this additional energy is in approximation constant, of the order of −15 eV (e.g., it corresponds to UV radiation of wavelength 88.6 nm). It can be in accordance with data by Laurencin [48], who estimated the energy differences between different Mg(I) and Mg(II) positions as ~0.1 eV. Considering the great difference in absolute levels of ΔE between Pb and Mg, the difference between Mg(I) and Mg(II) seems to be acceptable.

### 2.7. Discontinuities

**Figure 7:**
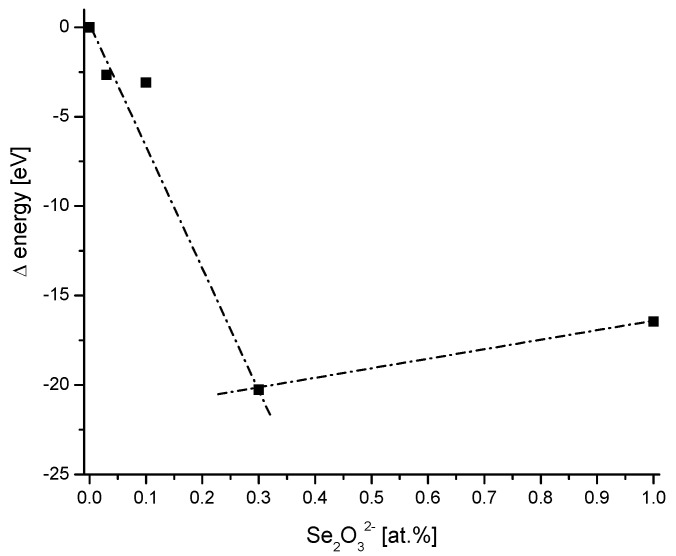
The involvement of Se in hydroxyapatite, data from [49]. Left side corresponds to substituted hydroxyapatite, the right one to the calcium selenite hydrate.

An interesting case can be observed in the introduction of selenites to the hydroxyapatite structure [49]. When we calculate the energy of the ion exchange of phosphates ions on selenites, the very clear discontinuity arrives in the relevant curve (Figure 7). It corresponds to an amount of 30 at% of Se_2_O_3_^2−^. The first increment of the curve corresponds to substituted hydroxyapatite. According to the authors, the hydroxyapatite transforms steadily in amorphous apatite and after the discontinuity point in calcium selenite hydrate. Therefore, the energy difference curve allows for precisely detecting the phase transition. 

## 3. Discussion

With very simple assumptions, mainly supported by the relationship λ = 12.4/E and by consistently putting the question of what the shift in the value of sinΘ means, we managed to derive several equations depicting the energy changes in apatite molecules. They were applied to solve the problem of energy changes associated with the ion exchanges inside the apatite/bioapatite molecules.

In the cases when the size of the ions corresponds roughly to Goldschmidt rules as applied to Ca ion as the reference, the involvement of foreign ions in the structure of apatites is possible. Sometimes it is going on smoothly, forming solid solutions. In our considerations, we divided the exchange locations in apatites, derived from the hydroxyapatite Ca_10_(PO_4_)_6_(OH)_2_ according to our coloristic notation on cations, anions, and ions existing in the tetrad channel. Then, the energies of substitutions were calculated using equations that were derived in this paper. Clear differentiation was observed. The cationic exchanges were going on with very different energy changes, which lead to new stable levels. It is surprising that the exchange of energy is so rigorously proportional to the d parameter for Bragg’s rule and to the crystallographic volume of the cell. Equations (11)–(13) can have significant meaning in the modelling of apatitic materials.

The anions are the next group, in which energy changes occur. 

The smallest energy changes are observed in the case of ion exchanges inside the tetrad channel. The above sequence is in part in accordance with old studies by Royce on the diffusion of ions inside apatites. According to his calculations [50], the transport of hydroxyl ions inside the channel demanded only 2 eV, while the transport in the transverse direction cost 10 eV. Similar values were presented in [51,52]. 

We are convinced that the postulated methods for the calculation of energy changes associated with ion exchanges inside apatites widen our understanding of those materials. They provide an objective method for the comparison of very variable ion substitutions with each other. For years, different scientists paid attention to meaningful differences between mineralogical and biological apatites. Here, we showed these differences at the energetic level. Indeed, the bioapatites obey relatively mild energetic changes (Figure 2b). The transformations demand the energy of the order of several electron volts; independently of the sign, however, it demands rather spending the energy. 

The variability of bioapatites is of great interest, at first from the medical point of view. Here, the changes in teeth were considered. After the maturation, further changes in tooth bioapatites mean saturation with carbonates and increasing the energetic state of the bioapatite. 

## 4. Materials and Methods

In present study, we presented theoretical analysis. For that reason using only XRD data is insufficient for our reasoning. The presence of clear shifts in diffraction peaks under consideration was the obligatory feature of such measurements. For the calculations, we use a wide review of scientific data about possible ion exchanges in apatites and bioapatites. There are some compilations of such data, e.g., by Pan and Fleet [53]. We found the most reliable sets of data as indicated in relevant parts of the article, in places where the data were applied. Sample raw data are presented in Supporting Materials S1.

The calculative part of our consideration was done with the Origin 9.1 program. The proposed calculations could be made only in a series of compounds of the same kind (e.g., for apatites) and existing in the same crystallographic structure (here the hexagonal structure for apatites), in general, for the isomorphic structures.

Raw data were added as a Supporting Materials.

## 5. Conclusions

The version of Bragg’s law in which the wavelength is substituted with energy can imply that the shifts in sinΘ values might correspond to changes in the energies of particles. If we have to use substances from one class of compounds (e.g., apatites) and existing in one crystallographic system (e.g., in a hexagonal one), then it is possible to derive the equations describing the energy changes in participating particles. In the case of ionic substitutions, the apatites and hydroxyapatites were divided into the essential cations, essential anions, and ions included in the tetrad channel. The changes in energies were calculated for each such group. It was very important that the energy changes were very rigorously joined with the ionic radii of cations. Moreover, the introduction of foreign cations resulted in the change of crystal cell volume strictly dependent on the ionic radius of new cation. Next, the changes of energy in bioapatites were determined by taking into consideration the level of concentrations of magnesium and carbonates. The non-uniformity of energetic states in apatites was considered with an example of hydroxyapatite where Ca was substituted with Pb, and a clear distinction in the energy level of Ca(I) and Ca(II) states was noticed. In addition, the variability of the energetic state of tooth apatites due to material aging was calculated. Finally, the introduction of selenite instead of phosphate groups was considered, where the discontinuity point indicated the phase transition. Our equations can be helpful and illustrative in consideration of different problems connected with apatites in bones, dentin, and enamel. With support from nanotechnology, new materials, and computer-aided design modeling, new solutions for biomedical applications can be developed.

## Data Availability

Not applicable.

## Supplementary Materials

The following are available online at https://www.mdpi.com/article/10.3390/molecules27248913/s1, Supplementary raw data and linear versions of Figure 4b,c are presented in Supporting Materials S1.

## Sample Availability

Samples of the compounds are not available from the authors.
