# Supplementary material for: Measurements of Energetic States Resulting from Ion Exchanges in the Isomorphic Crystals of Apatites and Bioapatites
Source: Molecules. 2022 Dec 15;27(24):8913. doi: 10.3390/molecules27248913 (PMC9781773; doi:10.3390/molecules27248913)
Supplement: Supplementary file 1 [file molecules-27-08913-s001.zip › molecules-2074185-supplementary.pdf]

Supplementary raw data

Mg (a,c, conc.)

9.36667	6.85536	0
9.22976	6.78393	20
9.11071	6.69762	40
9.02143	6.59643	60
8.89048	6.51905	80
8.72381	6.42679	100

Ba (a,c, conc)

9.48054	6.88108	0
10.00067	7.40473	60
10.07114	7.47568	64.7
10.1047	7.50946	72
10.18523	7.59054	80.7
10.23221	7.63784	86.7
10.28591	7.69189	92.7
10.30604	7.71216	96
10.32617	7.73243	100

Cd (a,c, conc.)

9.43	6.882	0
9.405	6.83	23.5
9.397	6.78	50.3
9.353	6.704	75.5
9.327	6.68	100

Cl (a,c, conc.)

9.42068	6.84237	0
9.42636	6.84473	12.5
9.42831	6.85775	25

9.43595	6.86485	37.5
9.43932	6.86722	50
9.4418	6.88142	75

F (a,c, conc.)

9.42028	6.87963	0
9.40083	6.88366	20
9.39257	6.88299	40
9.38333	6.88165	60
9.37556	6.88434	80
9.36972	6.88165	100

CO_3^{2-} B (a, c, conc.)

9.435	6.882	0
9.403	6.885	22
9.381	6.882	37
9.395	6.881	58
9.362	6.882	72
9.318	6.868	76

CO_3^{2-} A (a, c, conc.)

9.42	6.88	0
9.457	6.878	29.5
9.469	6.874	38.5
9.492	6.867	61.2
9.544	6.859	73.2

SeO_3^{2-} (a, c, conc.)

9.4089	6.8748	0
9.4081	6.8689	3
9.4124	6.8611	10

9.3895	6.8509	30
9.3968	6.8497	100

Age (age, a, c)

5	9.44977	6.88503
16.78571	9.4465	6.88522
29.28571	9.44582	6.88484
38.21429	9.44209	6.88646
43.57143	9.44107	6.88703
45.35714	9.43938	6.88275
59.28571	9.43203	6.88513
62.14286	9.43395	6.88513
70.71429	9.43284	6.89301
86.42857	9.43395	6.88494
87.85714	9.43588	6.88598

Δ E (Δr, ΔE, ΔV)

-28	-575	-107
-5	-138	-28
0	0	0
18	364	72
19	448	99
35	731	183

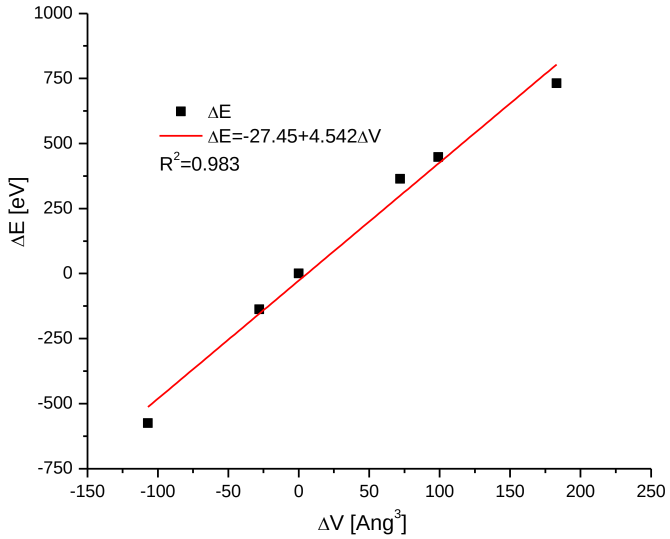

Linear version of Fig.4b

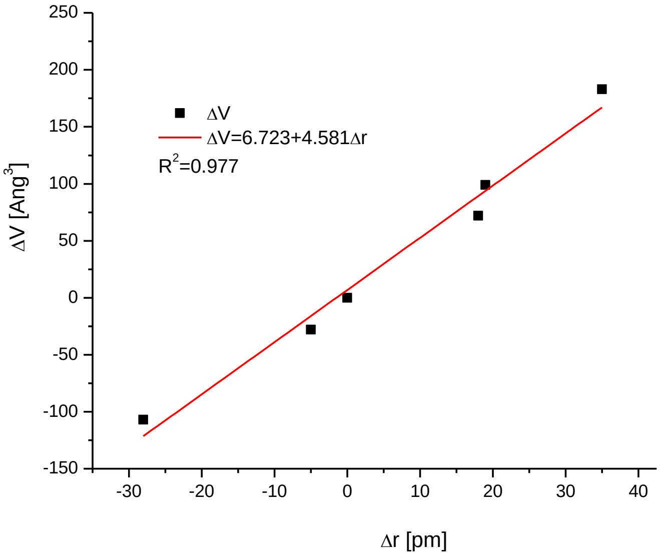

Linear version of Fig.4c
